# Population attributable fraction of incident HIV infections associated with alcohol consumption in fishing communities around Lake Victoria, Uganda

**DOI:** 10.1371/journal.pone.0171200

**Published:** 2017-02-16

**Authors:** Noah Kiwanuka, Ali Ssetaala, Ismail Ssekandi, Annet Nalutaaya, Paul Kato Kitandwe, Julius Ssempiira, Bernard Ssentalo Bagaya, Apolo Balyegisawa, Pontiano Kaleebu, Judith Hahn, Christina Lindan, Nelson Kaulukusi Sewankambo

**Affiliations:** 1 Department of Epidemiology and Biostatistics, School of Public Health, Makerere University, Kampala, Uganda; 2 Uganda Virus Research Institute-International AIDS Vaccine Initiative HIV Vaccine Program, Entebbe, Uganda; 3 Department of Medical Microbiology, School of Biomedical Sciences, Makerere University, Kampala, Uganda; 4 International AIDS Vaccine Initiative (IAVI), New York, New York, United States of America; 5 Medical Research Council/Uganda Virus Research Insitute, Uganda Research Unit on AIDS, Entebbe, Uganda; 6 Department of Infectious and Tropical Diseases, London School of Hygiene and Tropical Medicine, London, United Kingdom; 7 Department of Medicine, School of Medicine, University of California San Francisco, San Francisco, California, United States of America; 8 Makerere University College of Health Sciences, School of Medicine, Clinical Epidemiology Unit, Kampala, Uganda; University of Toronto Dalla Lana School of Public Health, CANADA

## Abstract

**Background:**

Although the association between alcohol consumption and HIV risk is well documented, few studies have examined the magnitude of new HIV infections that could be prevented by controlling alcohol use. We report the population attributable fraction (PAF) of incident HIV infections due to alcohol consumption among the HIV high-risk population of fishing communities along Lake Victoria, Uganda.

**Methods:**

In a community-based cohort study, 1607 HIV sero-negative participants aged 18–49 years were enrolled from eight fishing communities along Lake Victoria, Uganda. At follow up 12 months later, 1288 (80.1%) were seen and interviewed. At baseline and follow-up visits, participants completed interviewer-administered questionnaires on alcohol consumption, demographics, and sexual risk behavior, and were tested for HIV infection. HIV incidence and adjusted incident rate ratios (adjusted IRRs) were estimated using Poisson regression models; the crude and adjusted PAFs of incident HIV infections associated with alcohol consumption were calculated using the Greenland and Drescher method for cohort studies.

**Results:**

Among the 1288 participants seen at follow up, 53.5% reported drinking alcohol of whom 24.4% drank occasionally (2 days a week or less) and 29.1% drank regularly (3–7 days a week). Forty eight incident HIV infections occurred giving an incidence rate of 3.39/100 person years at-risk (pyar) (95% CI, 2.55–4.49). Compared to non-drinkers, the adjusted IRR of HIV was 3.09 (1.13–8.46) among occasional drinkers and 5.34 (2.04–13.97) among regular drinkers. The overall adjusted PAF of incident HIV infections due alcohol was 64.1 (95% CI; 23.5–83.1); ranging from 52.3 (11.9–74.2) among Muslims to 71.2 (32.6–87.7) for participants who reported ≥ 2 sexual partners in the past 12 months.

**Conclusion:**

In fishing communities along Lake Victoria, Uganda, 64% of new HIV infections can be attributed to drinking alcohol. Interventions to reduce alcohol consumption should be integrated in HIV/AIDS prevention activities for populations in whom both HIV and alcohol consumption are highly prevalent.

## Introduction

The association between alcohol consumption and HIV risk behaviours [1–16], and with prevalent and incident HIV infections are well documented [7,10,17–22,22,23]. Unhealthy alcohol consumption can increase the risk of HIV acquisition by two to five times compared to that among non-drinkers. HIV risk also rises with greater frequency of drinking [12,17,19,22,24]. Alcohol may influence HIV risk behavior through a variety of mechanisms: drinking can alter judgment and decision-making, reducing one’s perception of risk; venues where people drink may also be places where casual or transactional sex can easily be obtained; those who are intoxicated may forget about or be unable to use condoms [25–28]. Some emerging evidence from animal studies suggest that alcohol may increase biological susceptibility to HIV through effects on genital microbial flora [29] and immune system response [30], but these hypotheses are yet to be proven in humans. One study reported higher HIV viral loads in the genital mucosa of women reporting moderate to heavy alcohol consumption [31].

Unhealthy alcohol use appears to be a major factor fuelling the HIV epidemic in sub-Saharan Africa (SSA). In many countries in this region that also have generalized epidemics, per capita alcohol consumption is high [6,21,32–37]. However, little attention has been given to the idea of reducing alcohol consumption as a means to prevent the spread of HIV. Most countries do not mention alcohol reduction interventions in their HIV/AIDS control programs [33]. In addition, only a few studies on alcohol reduction for HIV prevention have been done in SSA, the majority of which demonstrated short term reductions in alcohol consumption but inconsistent changes in sexual behavior and HIV risk [6,34,35,38–40].

A paucity of literature exists on the magnitude of new HIV infections that could be prevented by reducing alcohol use in SSA. To our knowledge, only one study from Uganda has estimated the population attributable fraction (PAF) of incident HIV infections due to alcohol consumption; this study was conducted among female sex workers [24]. PAF measures the excess of disease or outcome that can be attributed to a given exposure thereby providing information on the proportional reduction in the outcome that would occur by eliminating the exposure, while keeping other other risk factors constant, assumming the exposure is causal. We estimated the proportion of new HIV infections that would be prevented in fishing communities along Lake Victoria, Uganda, if alcohol consumption were eliminated [41]. Fishing communities in Uganda have HIV prevalence and incidence rates that are five to seven times greater than those in the general population [17–19,42–45], and alcohol consumption has been shown to a major predictor of HIV infection in this population [17,19].

## Methods

### Study design and procedures

Between September 2011 and March 2013, we conducted a community-based longitudinal cohort study among adults recruited from eight fishing communities located in three districts bordering Lake Victoria (Wakiso, Mukono and Kalangala), Uganda. A fishing community was defined as a population living in a village or a trading center adjacent to the lake, either on the mainland or on an island, in which the main economic activities were directly or indirectly related to the fishing industry [46]. The population of these communities are heterogeneous and include fishermen; boat owners, makers and repairmen; fish processors and traders; shop keepers; owners and employees of bars/restaurants/lodges; sex workers; and housewives.

Detailed study procedures have been described previously [17,18,42]. Briefly, 2191 participants aged 18–49 years who had resided in fishing communities for at least six months prior to start of the study were enrolled after providing written informed consent. Those who were 18–49 years old were included because they are considered to be the most sexually active. During the community-based surveys, interviewer administered questionnaires were conducted in privacy by same sex interviewers and venous blood samples were collected for HIV testing. Among the 2191 baseline enrollees, 1607 were HIV sero-negative and were followed up to estimate the HIV incidence. Data on socio-demographic characteristics and self-reported male circumcision status were collected at the baseline visit only; information on alcohol consumption and sexual risk behaviors (number of sexual partners, new sexual partners, condom use) was collected at both baseline and follow up visits. Participants were asked if they drank any alcohol in the prior three and 12 months and if so, the frequency of consumption. Both three and 12-month recall periods were used in order to assess both short and long term alcohol consumption. In this article, we evaluated alcohol consumption in past 12 months because it was more likely to reflect drinking over the period during which HIV sero-conversion could have taken place, assuming that infection would have taken place in the mid-point between the baseline and follow-up visits. At each visit, participants received free HIV counseling and testing from certified HIV counselors. Those with symptoms of sexually transmitted infections (STIs) and men who were uncircumcised were referred to health facilities for treatment and voluntary medical male circumcision (VMMC), respectively. The protocol was reviewed and approved by the Uganda Virus Research Institute Research and Ethics Committee, and the Uganda National Council for Science and Technology.

### Laboratory testing

HIV status was determined by rapid HIV tests performed in the community by certified laboratory technologists, and enzyme linked immunoassay (EIA) with RNA PCR confirmation was done in the laboratory at the Uganda Virus Research Institute, Entebbe. Rapid diagnostic testing of HIV was performed according to the Uganda national testing algorithm. Blood samples were first tested with Alere Determine^®^ HIV-1/2 test (Alere Medical Co., Ltd., Chiba, Japan), and if non-reactive, results were reported as negative. Reactive samples were then tested with the HIV 1/2 STAT-PAK^®^ assay (Chembio Diagnostic Systems, Inc. Medford, NY, USA), and if reactive, results were reported as positive. If results were discordant on STAT-PAK^®^, Uni-Gold^™^ HIV test (Trinity Biotech plc, Bray, Ireland) was used as a tie-breaker. All samples positive on rapid tests were re-tested in duplicate using the Vironostika HIV Ag/Ab (Biomerieux, SA, Marcy-l’Etoile, France) EIA. Discordant EIA results were confirmed using HIV RNA PCR (COBAS^®^ AmpliPrep/COBAS^®^ TaqMan^®^ HIV-1 Test, v2.0, Roche Molecular Diagnostics, Pleasanton, CA, USA).

### Statistical analysis

Participants' socio-demographic characteristics were summarised and stratified by any alcohol consumption in the prior 12 months (none, any drinking) and by frequency of consumption (none, occasional, regular). Alcohol consumption was defined as "none" if a participant reported no consumption in the 12 months preceeding the date of the follow up visit, "occasional" if consumption was two days or less a week, and "regular" if they drank three to seven days per week. Chi-square tests were used to assess differences in proportions of participants’ socio-demographic and behavioral characteristics by levels of alcohol consumption. Multivariable Poisson regression models with empirical variance estimator [47] and the natural logarithm of pyar as the offset term, were used to estimate adjusted incidence rate ratios (adj.IRR) and their corresponding 95% confidence intervals. HIV seroconversion was estimated to have occurred at the midpoint between the baseline negative and follow up positive HIV tests. Person years-at-risk (pyar) were calculated as: (date of follow up seronegative sample or estimated date of HIV seroconversion minus date of the baseline seronegative sample) divided by 365.25. HIV incidence rates per 100 pyar were calculated as the number of events of seroconversion divided by pyar, multiplied by 100. The final multivariable model adjusted for sex, age, education, religion, tribe, occupation, duration of stay in the community, number of new sex partners, condom use and marital status. To estimate PAFs, alcohol consumption was modeled as a binary variable (any consumption versus none). The PAF of incident HIV infections associated with any alcohol consumption was calculated as [1-Pr(HIV incident infection | no alcohol consumption)/ Pr(HIV incident infection)] x100, using the Greenland and Drescher method for cohort studies [48]. This maximum likelihood method uses log transformation for normalization and variance-stabilization, and estimates the logs of two scenario means (conditional arithmetic mean outcomes), a baseline scenario (scenario 0) and an imagined scenario (scenario 1) in which one or more exposure variables are assumed to be set to particular values (typically zero), holding other predictor variables in the model constant. The method then it calculates the log of the ratio of scenario 1 mean to scenario 0 mean to estimate the PAF and uses the log transformed complement of PAF (log[1-PAF]) to estimate standard errors and 95% confidence intervals (CI) around the PAF [48,49]. Although lack of male circumcision was associated with a 2 fold increased risk HIV infection in unadjusted analyses, the association lost statistical significance at multivariable regression modeling and was excluded from the final model. However, we determined the PAFs associated with circumcision status by fitting a separate model for men only. A sensitivity analysis was conducted to assess the effect of the approximately 20% loss to follow up on the estimated of PAF. Multiple imputations were used impute missing data for the post baseline covariates. Multivariable logistic and Poisson regression were used to impute categorical and count covariates respectively. All statistical analyses were performed using Stata^®^ 14 (StataCorp, College Station, TX) software.

## Results

### Study population

Table 1 presents the baseline socio-demographic characteristics and follow up rates of the study population of 1607 HIV sero-negative participants enrolled at baseline. Fifty four percent (54.5%) of the participants were males, 43.1% were aged 30 years or more, about 80% were Christian, and only 35.4% had an education beyond primary school. Half of the participants were engaged in fishing or fishing related activities and 30.1% had spent less than 2 years in fishing communities. Eighty percent (1288) were followed up 12 months post-baseline. The reasons for loss to follow up (LTFU) included outmigration (50%), untraceable (46.3%), refusal to continue with the study (2.6%), and death (1.1%). The follow up rates did not differ statistically by sex, education level, religion or occupation. However, higher rates of follow up were observed among participants aged 30 years or more, the Baganda tribe (indigenous to the area), those who were married, and those who had spent five years or more in the fishing community.

**Table 1:** Study population Socio-demographic characteristics and follow up tates of fisherfolk who were HIV sero-negative at baseline (n = 1607).

	Baseline	Follow up (12 months)	
	Enrolled No. (%)	Followed No. (%)	Not followed No. (%)
**All Participants**	1607 (100)	1288 (80.1)	319 (19.9)
**Sex**			
Male	876 (54.5)	697 (79.6)	179 (20.4)
Female	731 (45.5)	591 (80.9)	140 (19.1)
**Age at enrolment (years)**			
30+	693 (43.1)	595 (85.9)	98 (14.1) ^††^
25–29	411 (25.6)	595 (79.6)	84 (20.4)
18–24	503 (31.3)	366 (72.8)	137 (27.2)
Highest education level*			
None	112 (7.0)	90 (80.4)	22 (19.6)
Primary	924 (57.6)	734 (79.4)	190 (20.6)
Post primary	568 (35.4)	461 (81.2)	107 (18.8)
**Religion**			
Muslim	327 (20.4)	263 (80.4)	64 (19.6)
Protestant/Evangelical	659 (41.0)	518 (78.6)	141 (21.4)
Roman Catholic	621 (38.6)	507 (81.6)	114 (18.4)
**Ethnicity/tribe**			
Non-Baganda	885 (55.1)	680 (76.8)	205 (23.2) ^††^
Baganda	722 (45.9)	608 (84.2)	114 (15.8)
**Occupation**			
Fishing/Fishing related	805 (50.1)	648 (80.5)	157 (19.5)
Trade/Business	165 (10.3)	135 (81.8)	30 (18.2)
Bar/Lodge/Restaurant	162 (10.1)	122 (75.3)	40 (24.7)
Farming	87 (5.4)	75 (86.2)	12 (13.8)
Housewife	129 (8.0)	101 (78.3)	28 (21.7)
Others^†^	259 (16.1)	207 (79.9)	52 (20.1)
**Duration in community at enrolment** (years)			
5 or more	709 (44.1)	650 (91.7)	59 (8.3) ^††^
2–4	414 (25.8)	333 (80.4)	81 (19.6)
Less than 2	484 (30.1)	305 (63.0)	179 (37.0)
**Marital status**			
Never married	305 (19.0)	214 (70.2)	91 (29.8) ^††^
Not currently married	330 (20.5)	263 (79.7)	67 (20.3)
Married monogamous	690 (42.9)	574 (83.2)	116 (16.8)
Married polygamous	282 (17.5)	237 (84.0)	45 (20.0)

*3 missing education,

^†^ Construction/Mechanic/Government/Clerical,

^††^Statistically significant at p<0.05.

### Alcohol consumption

Table 2 presents results on self-reported alcohol consumption and the frequency of drinking among the 1288 participants who contributed to the HIV incidence analysis. Slightly over half, 53.5%, reported drinking alcohol of whom 24.4% were occasional drinkers and 29.1% were regular drinkers. Among alcohol drinkers, 55.9% were male, 20.4% were Muslim, 55.6% were engaged in fishing/fishing related activities and 52.7% drank alcohol before sex. Those who were regular drinkers were more likely to have a greater number of sexual partners and to drink alcohol before sex. Condom use was lower among non-drinkers relative to occasional and regular drinkers.

**Table 2:** Alcohol consumption among the fishing communities of Lake Victoria, Uganda (n = 1288; HIV incidence analytic population).

	Any alcohol consumption in past 12 months			Frequency of alcohol consumption in past 12 months			
	No N (%)	Yes N (%)	P value	None N (%)	Occasional N (%)	Regular N (%)	P value
**All Participants**	599 (46.5)	689 (53.5)		599 (46.5)	314 (24.4)	375 (29.1)	
SOCIO-DEMOGRAPHICS							
**Sex**							
Male	290 (45.9)	408 (55.9)		290 (48.4)	146 (46.2)	262 (69.9)	
Female	309 (54.1)	281 (44.1)	<0.0001	309 (51.6)	168 (53.5)	113 (30.1)	<0.0001
**Age at enrolment (years)**							
30+	265 (47.5)	330 (49.8)		265 (44.2)	129 (41.1)	201 (53.6)	
25–29	145 (24.1)	182 (27.1)		145 (24.2)	93 (29.6)	89 (23.7)	
18–24	189 (28.4)	177 (23.1)	0.067	189 (31.5)	92 (29.3)	85 (22.7)	0.003
**Education**							
None/Primary	376 (45.6)	448 (54.4)		376 (45.6)	184 (22.3)	264 (32.)	
Secondary and above	221 (47.9)	240 (52.1)	0.426	221 (47.9)	129 (28.0)	111 (24.1)	0.005
**Religion**							
Muslim	175 (29.2)	88 (20.4)		175 (29.2)	47 (15.0)	41 (10.9)	
Protestant/Evangelical	255 (42.6)	263 (40.2)		255 (42.6)	124 (39.5)	139 (37.1)	
Roman Catholic	169 (28.2)	338 (39.4)	<0.0001	169 (28.2)	143 (45.5)	195 (52.0)	<0.0001
**Ethnicity/tribe**							
Non-Baganda	316 (52.8)	364 (52.8)	0.978	316 (52.8)	152 (48.4)	212 (56.5)	
Baganda	283 (47.2)	325 (47.2)		283 (47.2)	162 (51.6)	163 (43.5)	0.104
**Occupation**							
None fishing activities^†^	334 (55.8)	306 (44.4)		334 (55.8)	150 (47.8)	156 (41.6)	
Fishing/fishing related activities^††^	265 (44.2)	383 (55.6)	<0.0001	265 (44.2)	164 (52.2)	219 (58.4)	<0.0001
**Duration in community at enrollment** (years)							
5+	306 (51.1)	344 (49.9)		306 (51.1)	146 (46.5)	198 (52.8)	
2–4	156 (26.0)	177 (25.7)		156 (26.0)	88 (28.0)	89 (23.7)	
Less than 2	137 (22.9)	168 (24.4)	0.815	137 (22.9)	80 (25.5)	88 (23.5)	0.515
SEXUAL BEHAVIORS							
**Marital status**							
Not married	187 (39.2)	290 (42.1)		187 (39.2)	124 (39.5)	166 (44.3)	
Married	412 (50.8)	399 (57.9)	<0.0001	412 (50.8)	190 (60.5)	209 (55.7)	<0.0001
**New sex partners in past 12 months**							
0–1	494 (92.3)	486 (78.1)		494 (92.3)	241 (84.6)	245 (72.7)	
2+	41 (7.7)	136 (21.9)	<0.0001	41 (7.7)	44 (15.4)	92 (27.3)	<0.0001
**Condom use in past 12 months**							
No	373 (73.1)	328 (52.3)		373 (73.1)	153 (53.5)	175 (51.3)	
Yes	167 (26.9)	299 (47.7)	<0.0001	167 (26.9)	133 (46.5)	166 (48.7)	<0.0001
**Drank alcohol before sex**							
No	515 (87.6)	326 (47.3)		515 (87.6)	193 (61.5)	133 (35.5)	
Yes	73 (12.4)	363 (52.7)	<0.0001	73 (12.4)	121 (38.5)	242 (64.5)	<0.0001

^†^Farming, Shop keeping, Housewife, Construction/Mechanic/Government/Clerical, Fishing,

^††^Fishmongers, fish processing, boat maker/owner

### HIV incidence rate

Results on absolute HIV incidence rates and crude and adjusted rate ratios are presented in Table 3. Over a period of 12 months, 48 incident HIV infections occurred over 1416.8 pyar resulting in an overall incidence rate of 3.39/100 pyar (95% CI, 2.55–4.49). Of the 48 incident cases, 10 (20.8%) occurred among those who did not drink, 12 (25.0%) among occasional drinkers and 26 (54.2%) among regular drinkers, *p*<0.0001. Compared to non-drinkers, the adjusted IRR was 3.09 (95%: 1.13–8.46) for occasional drinkers and 5.34 (95% CI: 2.04–13.97) for regular drinkers. Among regular drinkers, the adjusted IRRs were significantly higher among males, persons aged 25–29 years, individuals involved in fishing and or fishing-related activities and those with less than two years duration of stay in fishing communities.

**Table 3:** Crude and adjusted HIV-1 Incidence rate ratios by frequency of alcohol drinking and population attributable fractions of new HIV infections due to alcohol consumption in fishing communities around Lake Victoria, Uganda (n = 1288).

	Incidence Rate (95% CI)	HIV-1 Incidence Rate Ratios (95% CI)						Population Attributable Fraction (95% CI)	
		Crude			Adjusted			Crude	Adjusted
Characteristic		Frequency of alcohol consumption			Frequency of alcohol consumption				
		None	Occasional	Regular	None	Occasional	Regular		
**All Participants**	3.39 (2.55–4.49)	1	2.33 (1.01–5.39) ^¶^	4.30 (2.07–8.92)^¶^	1	3.09 (1.13–8.46) ^¶^	5.34 (2.04–13.97)^¶^	55.8 (23.3–74.6)	64.1 (23.5–83.1)
**Sex**									
Male	3.40 (2.31–4.99)	1	2.00 (0.40–9.95)	7.72 (2.29–26.03)^¶^	1	2.92 (0.55–15.54)	11.11 (2.86–43.10)^¶^	58.2 (25.0–76.7)	66.1 (26.2–84.4)
Female	3.37 (2.22–5.13)	1	2.42 (0.90–6.50)	2.38 (0.80–7.07)	1	3.61 (1.06–12.34)	2.11 (0.40–11.27)	53.6 (11.4–75.6)	61.9 (20.8–81.6)
**Age at enrolment** (years)									
30+	2.45 (1.50–4.00)	1	1.54 (0.34–6.90)	3.06 (0.94–9.95)	1	5.29 (0.52–53.16)	7.78 (0.92–66.05)	57.3 (25.0–75.7)	65.3 (25.0–84.0)
25–29	4.77 (2.96–7.67)	1	4.02 (0.78–20.76)	8.44 (1.85–38.52)^¶^	1	4.67 (0.77–28.23)	11.35 (2.24–57.49)	57.2 (24.9–75.6)	64.2 (23.5–83.2)
18–24	3.68 (2.22–6.11)	1	2.10 (0.52–8.42)	4.04 (1.18–13.84)^¶^	1	2.86 (0.60–13.64)	3.37 (0.45–24.96)	53.7 (21.5–72.7)	63.0 (22.4–82.4)
**Education**									
None/Primary	3.23 (2.24–4.65)	1	1.76 (0.59–5.25)	3.33 (1.37–8.09)	1	3.18 (0.73–13.80)	4.45 (1.03–19.09)	54.8 (22.3–73.8)	65.2 (24.8–83.9)
Secondary and beyond	3.68 (2.35–5.77)	1	3.54 (0.88–14.18)	7.03 (1.93–25.52) ^¶^	1	3.05 (0.74–12.60)	5.80 (1.61–20.96)^¶^	54.9 (22.4–73.8)	62.7 (21.9–82.2)
**Religion**									
Muslim	2.06 (0.93–4.60)	1	2.48 (0.41–14.94)	1.47 (0.15–14.23)	1	4.67 (0.44–49.61)	2.48 (0.16–37.21)	38.8 (6.9–59.8)	52.3 (11.9–74.2)
Protestant/Evangelical	2.09 (1.18–3.67)	1	3.17 (0.53–18.97)	6.60 (1.37–31.81) ^¶^	1	5.29 (0.50–55.8)	12.35 (1.41–108.24)	49.0 (13.4–70.0)	62.3 (21.1–82.0)
Roman Catholic	5.44 (3.80–7.78)	1	1.67 (0.53–5.28)	3.24 (1.20–8.73) ^¶^	1	2.82 (0.76–10.47)	4.64 (1.40–15.42)	55.9 (19.3–75.9)	67.2 (26.7–85.3)
**Ethnicity/tribe**									
Baganda	2.52 (1.56–4.05)	1	2.97 (0.71–12.44)	5.34 (1.44–19.73) ^¶^	1	6.30 (1.10–35.98)	6.90 (1.32–36.18)	55.8 (23.3–74.5)	63.2 (22.3–82.6)
Non-Baganda	4.18 (2.94–5.95)	1	2.11 (0.74–6.02)	3.78 (1.57–9.11) ^¶^	1	2.76 (0.78–9.80)	5.22 (1.61–16.93)	55.9 (23.4–74.6)	64.6 (24.1–83.5)
**Occupation**									
None fishing activities^†^	2.96 (1.93–4.53)	1	2.25 (0.65–7.78)	4.84 (1.68–13.94) ^¶^	1	3.61 (0.81–15.96)	4.78 (0.93–24.47)	47.9 (16.2–67.5)	61.4 (20.7–81.4)
Fishing/fishing related activities^††^	3.82 (2.62–5.57)	1	2.31 (0.73–7.28)	3.77 (1.37–10.39) ^¶^	1	4.40 (1.14–16.95)	7.76 (2.08–28.96)	59.2 (26.7–77.3)	66.1 (25.8–84.5)
**Duration of stay in community** (years)									
5+	2.94 (1.91–4.51)	1	2.67 (0.71–9.94)	4.82 (1.55–14.97) ^¶^	1	3.31 (0.72–15.15)	3.90 (0.79–19.23)	55.3 (22.8–74.2)	64.7 (23.4–83.1)
2–4	3.00 (1.66–5.42)	1	0.88 (0.16–4.83)	2.21 (0.59–8.25)	1	2.01 (0.11–35.64)	5.40 (0.42–69.23)	55.8 (23.2–74.5)	65.3 (25.1–83.9)
Less than 2	4.75 (2.91–7.74)	1	4.45 (0.86–22.99)	7.40 (1.60–34.30) ^¶^	1	4.36 (0.68–28.01)	9.44 (1.80–49.44)	56.1 (23.5–74.8)	63.1 (22.1–82.5)
**Marital Status**									
Not Married	4.39 (2.92–6.61)	1	1.56 (0.45–5.40)	3.13 (1.11–8.78)	1	3.52 (0.71–17.37)	4.15 (0.86–20.06)	58.0 (25.0–76.5)	66.1 (25.8–84.5)
Married	2.80 (1.89–4.14)	1	3.06 (0.97–9.65)	5.21 (1.85–14.62)	1	5.22 (1.36–19.97)	8.50 (2.33–31.01)	53.2 (20.4–72.6)	62.2 (21.1–82.0)
**New sex partners in past 12 months**									
0–1	2.69 (1.87–3.87)	1	4.16 (1.42–12.18)^¶^	5.85 (2.11–16.26) ^¶^	1	3.52 (1.14–10.82) ^¶^	6.82 (2.36–19.66) ^¶^	53.3 (20.4–72.6)	61.4 (19.7–81.4)
2+	5.67 (3.14–10.23)	1	1.98 (0.18–21.92)	3.75 (0.47–30.08)	1	6.61 (2.28–19.20)	3.03 (0.90–10.19)	63.8 (31.6–80.9)	71.2 (32.6–87.7)
**Condom use**									
No	3.77 (2.62–5.42)	1	2.83 (1.03–7.79) ^¶^	4.47 (1.81–11.08) ^¶^	1	5.13 (1.25–21.02) ^¶^	7.18 (1.72–30.02) ^¶^	51.6 (18.7–71.1)	61.3 (19.7–81.4)
Yes	3.72 (2.37–5.83)	1	1.70 (0.38–7.64)	4.13 (1.16–14.68)^¶^	1	1.42 (0.34–5.89)	3.50 (0.93–13.22)	59.6 (26.6–77.7)	67.1 (27.1–85.2)

^†^Farming, Shop keeping, Housewife, Construction/Mechanic/Government/Clerical, Fishing,

^††^Fishmongers, fish processing, boat maker/owner,

^¶^ Statistically significant with p<0.05

### Population attributable fraction of incident HIV associated with alcohol consumption

The crude and adjusted PAFs of incident HIV infections due to alcohol consumption are also presented in the Table 3. After controlling for sex, age, religion, education, religion, tribe, occupation, duration of stay in fishing communities, marital status, new sexual partners, and condom use, the overall PAF of incident HIV due to alcohol consumption was 64.1% (95% CI; 23.5–83.1). The lowest adjusted PAF for alcohol use was observed among Muslims (52.3; 95% CI: 11.9–74.2) while the highest occurred among participants who reported two or more new sex partners in the past 12 months (71.2; 95% CI: 32.6–87.7). For all the covariates in the model, the estimated PAF increased after adjusting for confounders. In separate analyses that included males only, the adjusted PAF was 71.9 (95% CI: 6.9–91.5) among circumcised men and 79.3 (95% CI: 30.4–93.9) for those who were uncircumcised (data not shown).

### Sensitivity analysis of population attributable fraction after imputing missing data

Table 4 shows results of a sensitivity analysis that was conducted after imputing data for those lost to follow up. The estimated overall HIV incidence rate was 3.67/100 pyar (95% CI; 2.53–4.22) and did not statistically differ from the rate calculated from actual data (3.39/100 pyar (95% CI, 2.55–4.49). The adjusted PAF was 50.8 (95% CI; 19.0–70.1) overall; was higher in males [53.9 (95% CI; 21.8–72.8)], lowest among Muslims [37.2 (95% CI; 9.6–56.5)] and highest among participants who reported two or more new sexual partners in the past 12 months [58.2 (95% CI; 25.2–76.7)].

**Table 4:** HIV incidence and population attributable fractions of new HIV infections due to alcohol consumption using imputed data for those lost to follow up (n = 1607).

Characteristic	Incidence Rate (95% CI)	Population Attributable Fraction (95% CI)	
		Crude	Adjusted
**All Participants**	3.67 (2.53–4.22)	57.3 (31.2–73.5)	50.8 (19.0–70.1)
**Sex**			
Male	3.21 (2.26–4.57)	59.4 (33.2–75.4)	53.9 (21.8–72.8)
Female	3.33 (2.29–4.85)	54.6 (28.9–71.0)	47.3 (15.9–67.0)
**Age at enrolment** (years)			
30+	2.36 (1.49–3.75)	58.6 (32.4–74.7)	52.5 (20.3–71.7)
25–29	4.01 (2.53–6.36)	57.2 (30.8–73.5)	51.3 (19.3–70.6)
18–24	3.90 (2.58–5.91)	55.6 (30.0–71.8)	49.0 (17.7–68.5)
**Education**			
None/Primary	3.17 (2.29–4.39)	57.5 (31.1–73.8)	51.1 (19.2–70.5)
Secondary and beyond	3.45 (2.27–5.23)	57.1 (31.5–73.1)	50.3 (18.6–69.6)
**Religion**			
Muslim	2.20 (1.10–4.39)	45.4 (21.9–61.8)	37.2 (9.6–56.5)
Protestant/Evangelical	2.73 (1.77–4.22)	56.8 (30.9–73.0)	49.2 (17.0–68.9)
Roman Catholic	4.42 (3.09–6.32)	62.2 (35.9–77.7)	55.5 (22.3–74.5)
**Ethnicity/tribe**			
Baganda	2.48 (1.60–3.85)	57.7 (31.8–73.7)	50.3 (18.6–69.7)
Non-Baganda	3.92 (2.86–5.38)	57.1 (30.7–73.4)	51.1 (19.2–70.4)
**Occupation**			
None fishing activities^†^	2.91 (1.98–4.26)	55.2 (29.5–71.5)	47.9 (16.6–67.5)
Fishing/fishing related activities^††^	3.64 (2.57–5.13)	59.3 (32.9–75.3)	53.2 (21.0–72.3)
**Duration of stay in community** (years)			
5+	2.70 (1.76–4.15)	57.2 (30.8–73.5)	51.3 (19.5–70.6)
2–4	3.49 (2.14–5.68)	57.6 (31.6–73.7)	51.5 (19.5–70.7)
Less than 2	3.90 (2.55–5.96)	57.4 (31.5–73.5)	49.8 (18.0–69.3)
**Marital Status**			
Not Married	4.42 (3.12–6.28)	59.4 (33.2–75.3)	52.7 (20.5–71.9)
Married	2.51 (1.72–3.67)	55.9 (29.9–72.3)	48.6 (16.9–68.2)
**New sex partners in past 12 months**			
0–1	2.81 (2.08–3.80)	64.1 (38.1–79.2)	48.0 (16.2–67.7)
2+	5.69 (3.50–9.27)	55.8 (29.8–72.1)	58.2 (25.2–76.7)
**Condom use**			
No	3.00 (2.12–4.24)	54.2 (28.5–70.7)	47.2 (15.7–66.9)
Yes	3.67 (2.50–5.38)	61.3 (35.1–76.9)	55.3 (22.6–74.2)

^†^Farming, Shop keeping, Housewife, Construction/Mechanic/Government/Clerical, Fishing,

^††^Fishmongers, fish processing, boat maker/owner

## Discussion

In fishing communities along Lake Victoria, Uganda, we found that alcohol consumption was high and increased the risk of acquiring HIV infections by three to five times. In addition, we found that 64% of all new HIV infections could be attributed to drinking alcohol. Mathematically, this implies that discontinuing alcohol drinking in these communities would eliminate 64% of new HIV infections. This PAF of HIV due to alcohol was similar to what was estimated among sex workers in Kampala, Uganda [24]. Significantly reducing alcohol consumption would be a difficult task particularly when drinking is neither a cultural nor a religous taboo for the majority of residents. Even among Muslims in our study, whose faith prohibits alcohol drinking, one third reported some level of drinking. And one out of every five drinkers in this study was a Muslim. Alcohol regulation is also complicated, and involves legislation and enforcement of production, distribution, and sales, made particularly difficult when illegal ‘home-brew’ alcohol is widely available. Organizations that accrue monetary benefits from alcohol sales including government tax agencies, are likely to mount resistance [33]. In addition, light to moderate alcohol use has been shown to have cardioprotective and immune-boosting effects [50–53]. However, the cardioprotective effect may be due to genetic factors rather than alcohol consumption [54]. It is noteworthy that a study in South Africa found that the costs of addressing alcohol-related harms counterbalance the economic benefits obtained from alcohol sales hence justifying stricter alcohol regulation [55].

Regardless of the challenges to alcohol control, our findings show that effective interventions aimed at reducing alcohol consumption could play a big role in reducing the risk of HIV in fishing communities. A recent computer simulation study found that an intervention to reduce hazardous alcohol consumption in HIV-infected persons in East Africa could avert about 18,000 new infections and lead to considerable improvement in the quality-adjusted life years [56].

In this study, alcohol consumption was associated with HIV risk behaviours such as multiple sexual partners, high numbers of new sexual partners, and drinking alcohol before having sex; this was consistent with previous findings in Uganda [19,43], Tanzania, Kenya and Namibia [8]. Consistent condom use was higher among regular drinkers than occassional drinker and non-drinkers. This result is in same direction as the results of the study by Kiene *et al* which showed that being drunk during sex with casual partners was associated with increased condom use [15]. Despite the reported higher consistent condom use among regular drinkers, they had the highest HIV incidence rate which is paradoxical because consistent condom use should have led to protection against HIV acquisition [57]. While this observation could reflect reporting bias, the fact that drinkers also reported more sex partners (total and new) does not support the assumption. Qualitative findings from the Rakai cohort, Uganda, [44,58] and our unpublished data indicate that the high consumption of alcohol in fishing communities may be due to the abundance of drinking places, the availability of cheap locally made alcohol, the practice of easy-to-get easy-to-spend income from fishing, and the belief that alcohol makes it easier to endure fishing at night.

This study had several limitations. The primary goal of the study was to estimate HIV incidence and not to evaluate alcohol use. Thus, we did not use standardized alcohol questionnaires as such as the Alcohol Use Disorders Identification Test (AUDIT). However, our findings were similar to those reported in a previous study in Ugandan fishing communities that used the AUDIT interview[43]. Recall bias could have occurred for the self-report 12-months alcohol consumption given the length of the duration. There was statistically significant lower follow up rates participants aged 18–24 years and those not married yet both covariates were associated with lower levels of self-reported alcohol consumption, and this could have led to an overestimation of alcohol consumption levels. A sensitivity analysis conducted after imputing data for those lost to follow up showed a lower adjusted PAF [50.8 (95% CI; 19.0–70.1)] than that estimated from the actual data 64.1 (95% CI; 23.5–83.1). However, as observed from the actual data, the adjusted PAF was higher in males, lowest among Muslims, and highest among participants who reported two or more new sexual partners in the at-risk interval. Finally, we did not control for STIs due to the lack of data. However, a study done in Rakai, Uganda, showed that the PAFs of incident HIV due to symptomatic STIs was less than 10% [59]. A major strength of our study was using a random sample of fisherfolk from three different communities which increases the likelihood of representativeness of fisherfolk in this region.

### Conclusion

In fishing communities along Lake Victoria, Uganda, the PAF of incident HIV infections associated with alcohol consumption is 64%. Interventions to reduce alcohol consumption are urgently needed as part of HIV/AIDS prevention.
